# Supplementary material for: The interactome of intact mitochondria by cross-linking mass spectrometry provides evidence for coexisting respiratory supercomplexes
Source: Mol Cell Proteomics. 2017 Dec 8;17(2):216–32. doi: 10.1074/mcp.RA117.000470 (PMC5795388; doi:10.1074/mcp.RA117.000470)
Supplement: Supplemental Data [file supp_RA117.000470_133922_0_supp_23978_fzffwf.zip › spectra_annotation/mito_DR_spectra_annotation/465-1-14-1-11-1.pdf]

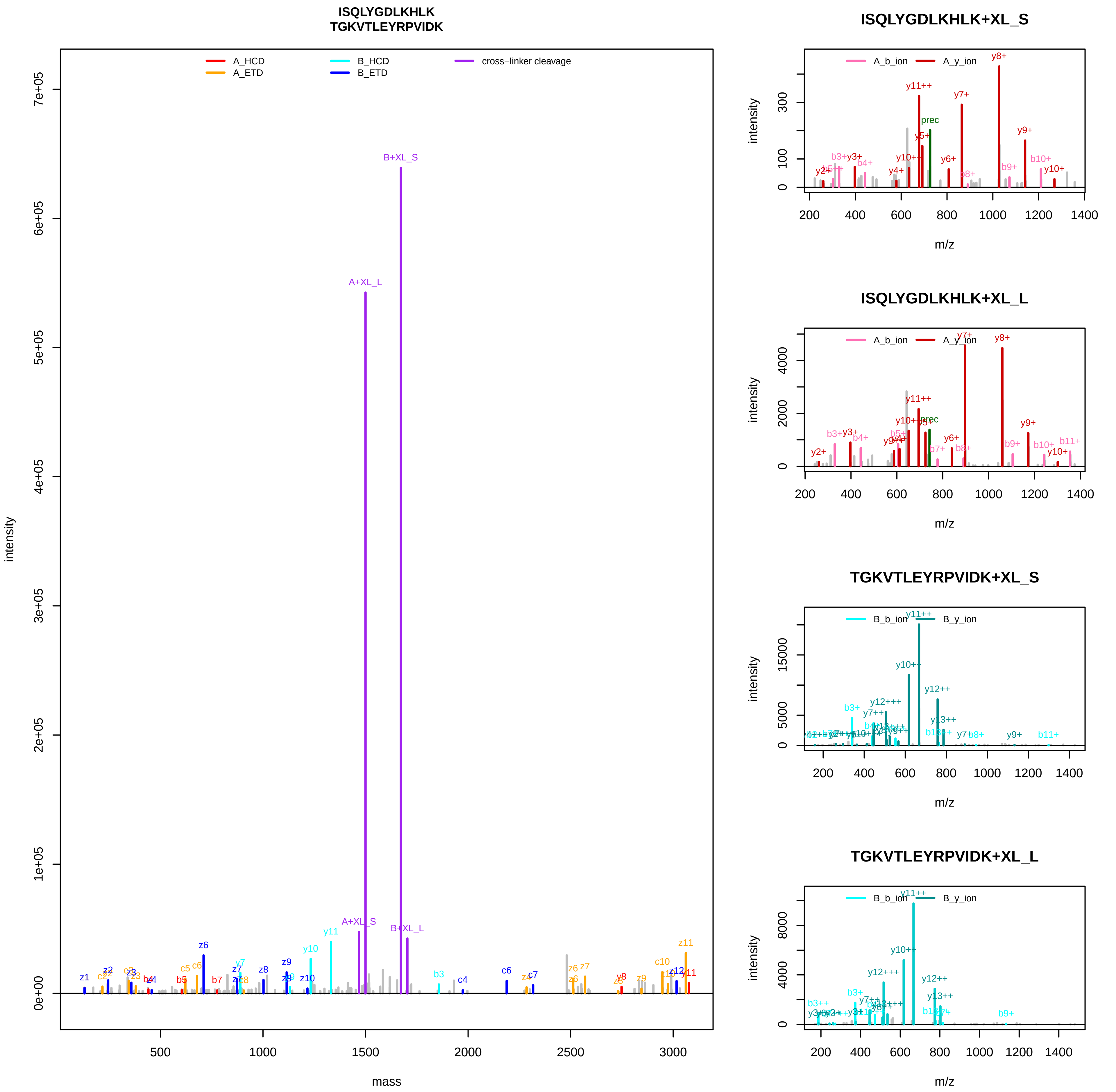
ISQLYGDLKHLK
TGKVTLEYRPVIDK
A_HCD
A_ETD
B_HCD
B_ETD
cross−linker cleavage
B+XL_S
A+XL_L
A+XL_S
B+XL_L
intensity
mass
0e+00
1e+05
2e+05
3e+05
4e+05
5e+05
6e+05
7e+05
500
1000
1500
2000
2500
3000
z1
z2
z3
z4
z6
z7
z8
z9
z10
z12
c4
c5
c6
c7
c10
b3
b4
b5
b7
y7
y8
y10
y11
z11
ISQLYGDLKHLK+XL_S
ISQLYGDLKHLK+XL_L
TGKVTLEYRPVIDK+XL_S
TGKVTLEYRPVIDK+XL_L
A_b_ion
A_y_ion
B_b_ion
B_y_ion
prec
y8+
y7+
y11++
y9+
y6+
y10++
y10+
b9+
b10+
b11+
y12++
y12+++
y13++
m/z
200
400
600
800
1000
1200
1400
0
100
300
2000
4000
5000
15000
8000
